# Low protein diets produce divergent effects on energy balance

**DOI:** 10.1038/srep25145

**Published:** 2016-04-28

**Authors:** Adel Pezeshki, Rizaldy C. Zapata, Arashdeep Singh, Nicholas J. Yee, Prasanth K. Chelikani

**Affiliations:** 1 Department of Production Animal Health, Faculty of Veterinary Medicine, University of Calgary, Calgary, Alberta, Canada; 2 Gastrointestinal Research Group, Snyder Institute for Chronic Diseases, University of Calgary, Calgary, Alberta, T2N 4N1, Canada

## Abstract

Diets deficient in protein often increase food consumption, body weight and fat mass; however, the underlying mechanisms remain poorly understood. We compared the effects of diets varying in protein concentrations on energy balance in obesity-prone rats. We demonstrate that protein-free (0% protein calories) diets decreased energy intake and increased energy expenditure, very low protein (5% protein) diets increased energy intake and expenditure, whereas moderately low protein (10% protein) diets increased energy intake without altering expenditure, relative to control diet (15% protein). These diet-induced alterations in energy expenditure are in part mediated through enhanced serotonergic and β-adrenergic signaling coupled with upregulation of key thermogenic markers in brown fat and skeletal muscle. The protein-free and very low protein diets decreased plasma concentrations of multiple essential amino acids, anorexigenic and metabolic hormones, but these diets increased the tissue expression and plasma concentrations of fibroblast growth factor-21. Protein-free and very low protein diets induced fatty liver, reduced energy digestibility, and decreased lean mass and body weight that persisted beyond the restriction period. In contrast, moderately low protein diets promoted gain in body weight and adiposity following the period of protein restriction. Together, our findings demonstrate that low protein diets produce divergent effects on energy balance.

The consumption of protein triggers adaptive responses in ingestive behavior, energy expenditure and metabolism that are under homeostatic controls. The ‘protein leverage’ hypothesis posits that protein intake is tightly regulated in several species including rats, mice and humans, which consume to acquire their required protein rather than meet requirements for fats and carbohydrates^1^,^2^,^3^. An increase in dietary protein density would decrease intake of carbohydrates and fats with consequent reduction in energy intake. There is substantial evidence that high protein diets promote satiety, weight loss and improve glycemic control^4^,^5^. A corollary to this hypothesis is that a reduction in the dietary protein concentration would increase total energy intake, due to overconsumption of carbohydrates and fat, in an effort to meet protein requirements. Consistent with this, moderately protein deficient diets were found to produce hyperphagia in rodents^6^,^7^,^8^,^9^ and in some^1^,^10^,^11^ but not all human studies^12^,^13^, whereas, severe protein restriction below a certain threshold leads to a reduction in food intake in rodents^9^,^14^. However, little is known of the underlying mechanisms by which moderate protein deficiency elicits such behavioural and metabolic adaptations and promotes positive energy balance with consequent predisposition to obesity and other metabolic disorders.

The hyperphagic effects of moderately low protein diets are purported to be through multiple mechanisms. These include imbalances in plasma and brain amino acid concentrations in rats^15^,^16^,^17^, modulation of energy sensors in the hypothalamus and anterior piriform cortex in rats^18^, and increased activity in the reward areas such as the orbitofrontal cortex and striatum in humans^11^. Low protein diets also enhance energy expenditure in rodents^17^,^19^,^20^,^21^,^22^, however, the underlying mechanisms are poorly understood. Potential mechanisms include increased sympathetic flux via β-adrenergic receptor (β-AR) signaling to brown adipose tissue (BAT) with consequent upregulation of mitochondrial uncoupling protein-1 (UCP1) expression^19^,^21^,^22^,^23^, as well as increased fibroblast growth factor-21 (FGF21) mediated thermogenesis^17^. However, the relative importance of these mechanisms, and whether gut-derived signals are associated with low protein induced hyperphagia and thermogenesis, are largely unknown.

The enteroendocrine cells of the gut secrete multiple hormones including peptide YY (PYY) which is postulated to play a role in the anorexigenic effects of high protein diets^24^; it is unknown whether gut hormones mediate the effects of low protein diets on energy balance. Apart from these hormones, gut-derived serotonin, which accounts for over 95% of total body serotonin^25^, has recently been shown to induce obesity due in part to reduced brown fat thermogenesis^26^,^27^, whereas brain-derived serotonin was reported to induce thermogenesis in brown fat^28^. The effects of serotonin are mediated by multiple receptor subtypes, of which 5-hydroxytryptamine (5HT3) receptors mediate the hypophagic effects of carbohydrate and fat^29^,^30^,^31^. It is unknown whether 5HT3 receptors mediate the effects of low protein diets on energy balance.

In addition to modulating energy intake and expenditure, moderate protein restriction in rats and mice has been consistently associated with alterations in body composition including reduction in lean mass, increased body fat content and development of fatty liver^6^,^8^,^9^,^14^,^17^,^32^,^33^. Protein restricted rats attempt to catch-up on body weight and adipose reserves following refeeding on a standard protein diet^34^,^35^,^36^,^37^,^38^. However, less is known of the temporal changes in body composition, energy intake and energy expenditure following realimentation of protein restricted animals to standard protein diets. Therefore, in this study, we investigated the effects of low protein diets varying in protein density on multiple metabolic parameters in obesity-prone rats. We demonstrate that protein deprivation decreased energy intake and increased energy expenditure. We also show that low to moderate protein deficiency increased energy intake with either an increase or no change in energy expenditure. Importantly, the effects on energy expenditure are mediated through enhanced serotonergic and β-AR signaling with concomitant upregulation of key thermogenic markers in BAT and skeletal muscle. Further, in contrast to the decreased lean mass and increased hepatic lipidosis observed with protein starvation or low protein diets, we provide evidence that moderate protein deficiency promoted gain in body weight and adipose reserves following the period of dietary protein restriction.

## Results

### Energy Intake, Energy Expenditure and Energy Digestibility

**Figure 1:**
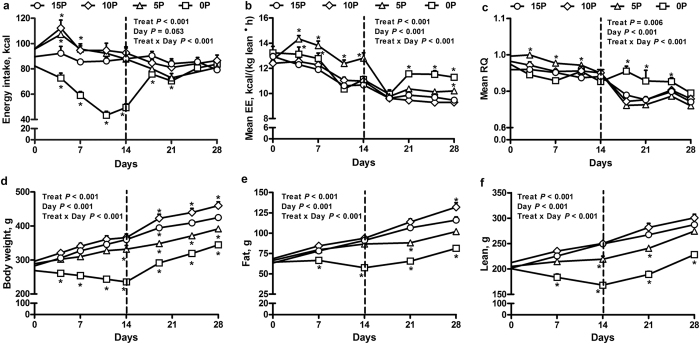
Effect of low protein diets on energy balance. (**a**) Daily energy intake, (**b**) mean energy expenditure (EE), (**c**) mean respiratory quotient (RQ), (**d**) body weight, (**e**) body fat mass and (**f**) body lean mass of obesity-prone rats. The animals were fed either a control (15% protein; 15P), moderately low protein (10% protein; 10P), very low protein (5% protein; 5P) or protein-free (0% protein; 0P) isocaloric diet for 14 days, followed by a realimentation phase with ad libitum access to the control diet (15P) for another 14 days. Dotted line separates the restriction and recovery phases. Values are mean ± SEM, n = 13–16. *P < 0.05 *vs* 15P.

When compared to the daily energy intake of rats fed a control diet (15% protein, 15P; see Supplementary Table S1), in rats fed a protein-free diet (0P), energy intake was decreased by 13–49% during the 14-day restriction and by 14–54% during the 21-day restriction, with subsequent recovery to control levels after the first week of realimentation (Fig. 1a, see Supplementary Figs S1a,S2a,b and S3a–h). Relative to 15P, daily energy intake was increased by 12–16% and 11–21% during the first 7 days in rats fed very low protein diet (5% protein; 5P) and moderately low protein diet (10% protein; 10P), respectively (Fig. 1a, see Supplementary Figs S2a,b and S3a–d). Compared to 15P, the mean daily energy expenditure was increased by 7% for 7 days in 0P and by 16–20% for 14 days in the 5P (Fig. 1b, see Supplementary Figs S2c,d and S4a,b). The increased energy expenditure of 0P and 5P was persistent even when lean mass was used as a covariate (see Supplementary Fig. S2g–i). Relative to 15P, respiratory quotient (RQ) was decreased during restriction in 0P (see Supplementary Fig. S1c) but increased in the 5P from day (d) 4 until d 11 (Fig. 1c). Further, by d 7, relative to 15P, total energy digestibility was increased by 16% in 10P but decreased by 41% in 0P. By d 14, energy digestibility tended (*P* < 0.1) to be decreased by 12% in 5P and decreased by 42% in 0P compared with 15P (Supplementary Table S2). Interestingly, when the 0P rats were realimented to the 15P diet, the mean daily and dark period energy expenditure was increased from d 21 onwards, and mean daily RQ also increased from d 18 until d 25 (Fig. 1b,c, see Supplementary Figs S1b, S2c,d and S4e–h).

### 5HT3 receptor blockade with Ondansetron

**Figure 2:**
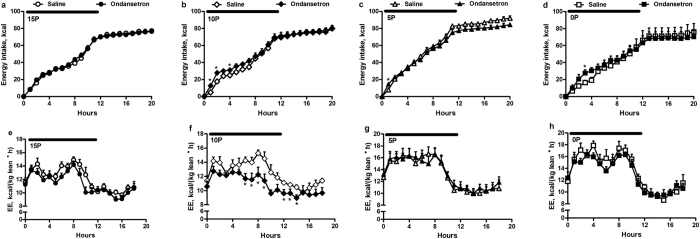
Effect of ondansetron on energy balance of rats fed low protein diets. Energy intake (**a–d**) and energy expenditure (EE; (**e–h**) of obesity-prone rats fed either a control (15% protein; 15P), moderately low protein (10% protein; 10P), very low protein (5% protein; 5P) or protein-free (0% protein; 0P) isocaloric diet with injections of saline or ondansetron (1.0 mg/kg; IP). The analyses of data from the first 9 hours of dark period revealed that there were significant fixed effects of dietary treatment (*P* < 0.001), drug (*P* = 0.019) and time (*P* < 0.001) and interactions of dietary treatment × time (*P* < 0.001) and drug × time (*P* = 0.046) for calorie intake. Similarly, there were significant effects of time and dietary treatment (*P* < 0.001) and interaction of dietary treatment × time (*P* < 0.001) for energy expenditure. Values are mean ± SEM, n = 8. *P < 0.05 saline *vs* ondansetron.

Ondansetron, a selective 5HT3 receptor blocker, was used to determine whether the serotonergic system mediates the effects of low protein diets on energy intake and expenditure. Ondansetron increased energy intake by 25–58% for the first 4 hours of dark period in 10P, by 82% at 1 hour (h) in 5P and by 68% at 3 h in 0P but not in 15P rats (Fig. 2a–d). Relative to vehicle, ondansetron reduced energy expenditure by 11–23% from 6 to 14 h in 10P, but not in 0P, 5P and 15P (Fig. 2e–h).

### β-AR blockade with Propranolol

**Figure 3:**
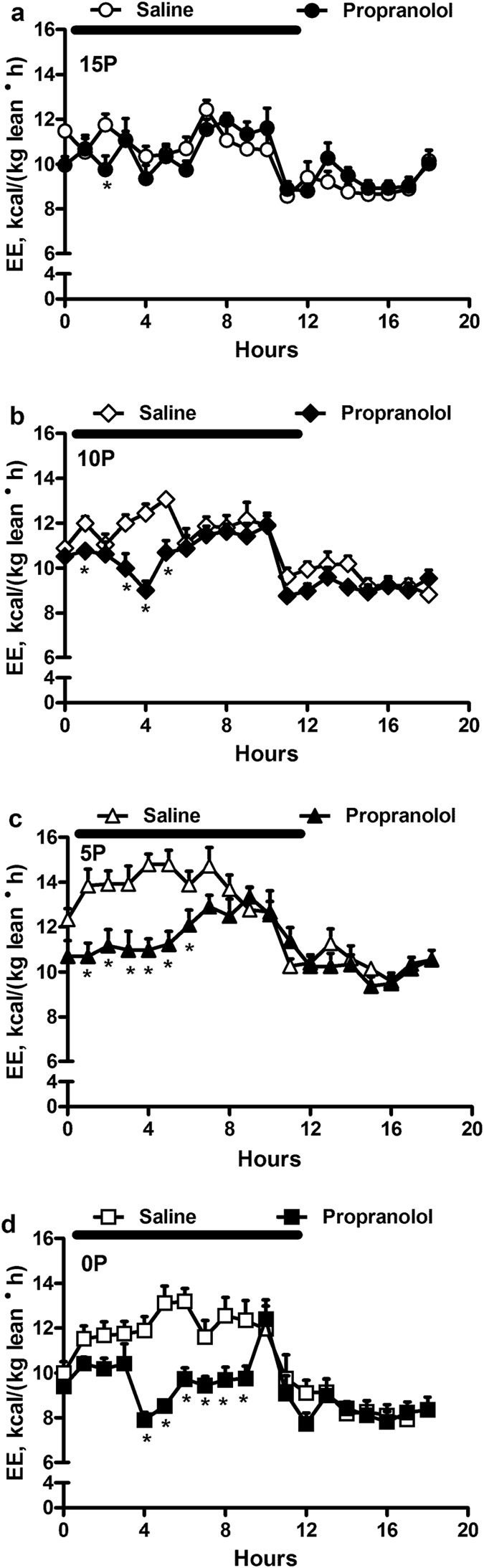
Effect of propranolol on energy expenditure. Obesity-prone rats were fed either a control (15% protein; 15P), moderately low protein (10% protein; 10P), very low protein (5% protein; 5P) or protein-free (0% protein; 0P) isocaloric diet with injections of saline or propranolol (10 mg/kg; SC). The analyses during the first 8 hours of dark period revealed that there were significant effects of time, drug, dietary treatment (*P* < 0.001), dietary treatment × time (*P* = 0.002), drug × time (*P* < 0.001), dietary treatment × drug (*P* = 0.026) and dietary treatment × drug × time (*P* = 0.009) for energy expenditure (EE). Values are mean ± SEM, n = 8. *P < 0.05 saline *vs* propranolol.

To determine whether the sympathetic system mediates the effects of low protein diets on energy expenditure, we administered propranolol, a β1 and β2-AR blocker. Propranolol decreased energy expenditure in the 0P, 5P and 10P rats during the dark period by 18%, 14% and 8%, respectively (Fig. 3a–d). Further, area under the curve (AUC) analyses revealed that propranolol decreased energy expenditure to a greater extent in 0P (10%) and 5P (8%) than 15P.

### Body composition

Relative to 15P rats, the body weight of 0P decreased by 15–35% during the 14-day restriction, by 18–42% during the 21-day restriction, and remained lower by 19–26% during realimentation (Fig. 1d, see Supplementary Fig. S1d). Similarly, the body weight of 5P decreased by 8% by d 14 and remained lower by 8–12% compared to 15P during realimentation. The body weight of 10P did not change during restriction but increased by 7–8% during realimentation. Body composition analyses revealed that, relative to 15P, the fat mass of 0P decreased by 15–37% during the 14-day restriction, by 18–48% during the 21-day restriction, and was also reduced by 28–38% during realimentation (Fig. 1E, see Supplementary Figs S1e and S2e). During the 14-day restriction, though the fat mass of 5P and 10P were similar to 15P (Fig. 1e), the 5P had relatively greater fat% (see Supplementary Fig. S2e). During realimentation, the fat mass of 5P decreased by 17% on d 21 whereas the fat mass of 10P increased by 11% by d 28 (Fig. 1e); fat% also followed a similar pattern (see Supplementary Fig. S2e). The lean mass of 0P decreased by 19–33% during the 14-day restriction, by 18–39% during the 21-day restriction, and remained lower by 21–29% during realimentation when compared to 15P (Fig. 1f, see Supplementary Figs S1f and S2f). Relative to 15P, in 5P lean mass was 12% and 10% lower by d 14 and d 21, respectively (Fig. 1f), but lean% were relatively greater in 0P and 5P during realimentation (see Supplementary Fig. S2f). When expressed as proportions of body weight, the 0P had 26% greater heart weight, while 5P had 15% greater liver weight compared to 15P (Supplementary Table S2). Further, the 5P and 10P had 58% and 16% greater liver fat%, and the 0P tended (*P* = 0.06) to have 26% greater liver fat%, than 15P (Supplementary Table S2). Compared to 15P, the efficiency of converting energy consumed to body weight was greater in the 0P during d 8–14 of restriction and throughout realimentation, whereas the efficiency was greater for 5P and 10P during d 22–28 of realimentation (see Supplementary Table S4).

### Plasma amino acids, glucose and hormones

**Figure 4:**
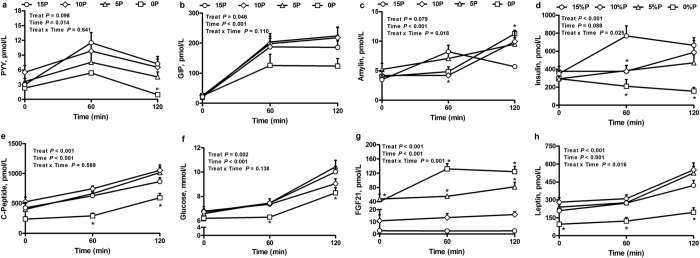
Effect of low protein diets on plasma hormone concentrations. (**a**) Peptide YY (PYY), (**b**) glucose-dependent insulinotropic peptide (GIP), (**c**) amylin, (**d**) insulin, (**e**) C-Peptide, (**f**) glucose, (**g**) fibroblast growth factor 21 (FGF21) and (**h**) leptin concentrations in obesity-prone rats. The animals were fed either a control (15% protein; 15P), moderately low protein (10% protein; 10P), very low protein (5% protein; 5P) or protein-free (0% protein; 0P) isocaloric diet for 14 days. Values are mean ± SEM, n = 5–9. *P < 0.05 *vs* 15P.

The 0P, 5P and 10P had lower postprandial plasma concentrations of the essential amino acids - threonine, tryptophan, valine, phenylalanine, leucine, isoleucine and lysine compared with 15P rats (see Supplementary Table S3). The concentration of histidine was decreased in 5P and 10P but not 0P, arginine was increased in 0P and methionine was decreased in the 0P and 5P. For non-essential amino acids, 0P, 5P and 10P had greater concentrations of serine and alanine, 0P and 5P had greater glycine but lower tyrosine, and 0P had lower ornithine, relative to 15P. Plasma concentrations of PYY, leptin, insulin, C-peptide and blood glucose were decreased, and amylin and glucose-dependent insulinotropic peptide (GIP) tended (*P* < 0.1) to be decreased, following a meal in 0P compared to 15P (Fig. 4). Plasma insulin concentrations in 5P and 10P, and glucose in 10P, were transiently decreased compared to 15P. Importantly, plasma FGF21 concentrations were increased in 0P and 5P, and tended in 10P (*P* < 0.1). Blood glucose concentrations and total glucose AUC following an intraperitoneal glucose tolerance test (IPGTT) did not differ among treatment groups (see Supplementary Fig. S5a,b).

### The mRNA and protein abundance of key molecules of energy metabolism in liver

**Figure 5:**
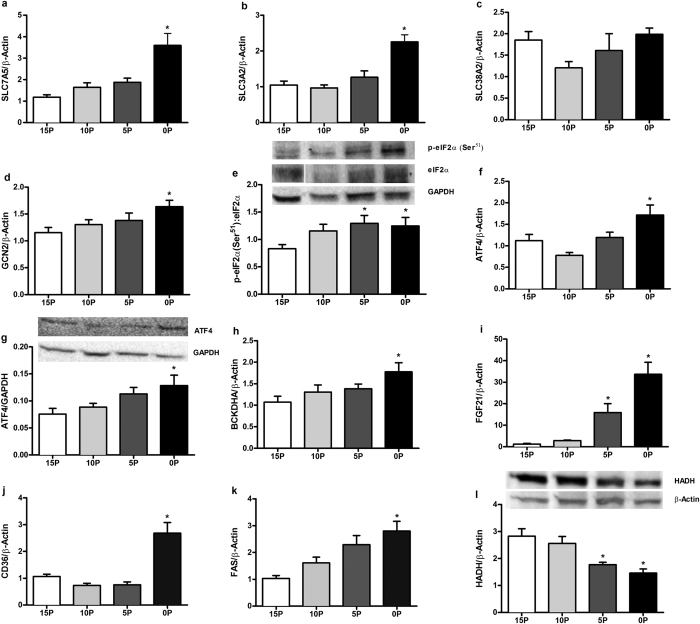
Effects of low protein diets on relative mRNA or protein abundance of key regulatory molecules of energy metabolism in liver. (**a**) Solute carrier family 7 member 5 (SLC7A5), (**b**) solute carrier family 3 member 2 (SLC3A2), (**c**) solute carrier family 38 member 2 (SLC38A2), (**d**) general control non-depressible 2 (GCN2), (**e**) serine 51 phosphorylated eukaryotic initiation factor 2α (peIF2α (Ser^51^)):eIF2α ratio, (**f,g**) activating transcription factor 4 (ATF4), (**h**) branched chain keto acid dehydrogenase E1, alpha polypeptide (BCKDHA), (**i**) fibroblast growth factor 21 (FGF21), (**j**) cluster of differentiation 36 (CD36), (**k**) fatty acid synthase (FAS), (**l**) 3-hydroxyacyl-CoA dehydrogenase (HADH) in obesity-prone rats. The animals were fed a control (15% protein; 15P), moderately low protein (10% protein; 10P), very low protein (5% protein; 5P) or protein-free (0% protein; 0P) isocaloric diet for 14 days. The relative mRNA and protein abundance was determined by qPCR and immunoblot analysis. β-Actin or glyceraldehyde 3-phosphate dehydrogenase (GAPDH), were used as reference targets. The eIF2α, peIF2α (Ser^51^), and GAPDH were reprobed on the same blots. Values are mean ± SEM, n = 5–9. *P < 0.05 *vs* 15P.

The mRNA abundance of molecules involved in amino acid uptake - solute carrier family 7 member 5 (SLC7A5), solute carrier family 3 member 2 (SLC3A2), amino acid sensing - general control non-depressible 2 (GCN2) and activating transcription factor 4 (ATF4), amino acid metabolism - branched chain keto acid dehydrogenase E1, alpha polypeptide (BCKDHA), fatty acid uptake - cluster of differentiation 36 (CD36), and fatty acid synthesis - fatty acid synthase (FAS) were all greater in 0P, and FAS tended (*P* < 0.1) to be greater in 5P, compared to 15P rats (Fig. 5a,b,d,f,h,j,k). Relative to 15P, the protein abundance of amino acid sensing molecules such as ATF4 in 0P and serine-51 phosphorylated eukaryotic initiation factor 2α (peIF2α (Ser^51^)):eIF2α ratio in 0P and 5P were greater, and the abundance of β-oxidation enzyme 3-hydroxyacyl-CoA dehydrogenase (HADH) were lower in 0P and 5P (Fig. 5e,g,l). Further, the 0P and 5P had greater mRNA abundance of FGF21 than 15P (Fig. 5i).

### The mRNA abundance of key molecules of energy metabolism in BAT

**Figure 6:**
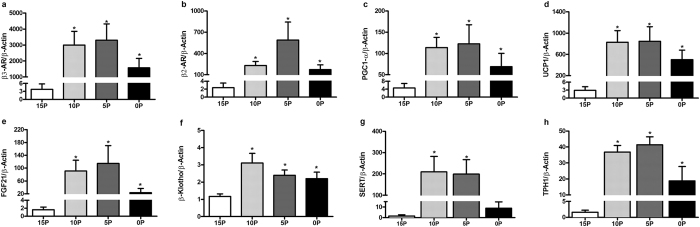
Effects of low protein diets on relative mRNA abundance of key regulatory molecules of thermogenesis in interscapular brown adipose tissue. (**a**) β3-adrenergic receptors (β3-AR), (**b**) β2-adrenergic receptors (β2-AR), (**c**) peroxisome proliferator-activated receptor gamma coactivator 1 α (PGC1-α), (**d**) uncoupling protein 1 (UCP1), (**e**) fibroblast growth factor 21 (FGF21), (**f**) β-Klotho, (**g**) serotonin transporter (SERT) and (**h**) tryptophan hydroxylase 1 (TPH1) in obesity-prone rats. The animals were fed either a control (15% protein; 15P), moderately low protein (10% protein; 10P), very low protein (5% protein; 5P) or protein-free (0% protein; 0P) isocaloric diet for 14 days. The relative mRNA abundance was determined by qPCR using β-Actin as reference target. Values are mean ± SEM, n = 5–9. *P < 0.05 *vs* 15P.

When compared to 15P rats, 0P, 5P and 10P had greater mRNA abundance of thermogenic genes encoding for β3-AR, β2-AR, peroxisome proliferator-activated receptor gamma coactivator 1 α (PGC1-α), UCP1, FGF21 and its co-receptor β-Klotho (Fig. 6a–f). Further, the transcript abundance of a key enzyme in serotonin synthesis - tryptophan hydroxylase 1 (TPH1) was increased in 0P, 5P, and 10P, and the serotonin reuptake transporter (SERT) was also increased in 5P and 10P, relative to 15P (Fig. 6g,h).

### The mRNA abundance of key molecules of energy metabolism in muscle

**Figure 7:**
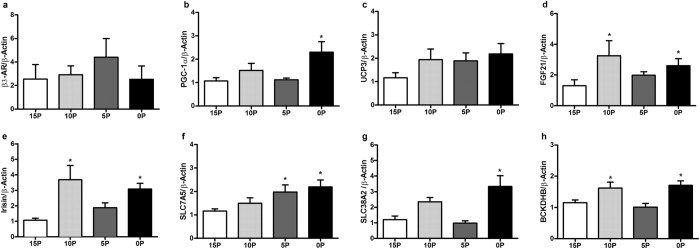
Effects of low protein diets on relative mRNA abundance of key regulatory molecules of energy metabolism in skeletal muscle. (**a**) β3-AR (β3-adrenergic receptors), (**b**) peroxisome proliferator-activated receptor gamma coactivator 1 α (PGC1-α), (**c**) uncoupling protein 3 (UCP3), (**d**) fibroblast growth factor 21 (FGF21), (**e**) irisin, (**f**) solute carrier family 7 member 5 (SLC7A5), (**g**) solute carrier family 38 member 2 (SLC38A2) and (**h**) branched chain keto acid dehydrogenase E1, beta polypeptide (BCKDHB) in obesity-prone rats. The animals were fed either a control (15% protein; 15P), moderately low protein (10% protein; 10P), very low protein (5% protein; 5P) or protein-free (0% protein; 0P) isocaloric diet for 14 days. The relative mRNA abundance was determined by qPCR using β-Actin as reference target. Values are mean ± SEM, n = 5–9. *P < 0.05 *vs* 15P.

In the skeletal muscle, the mRNA abundance of thermogenic genes such as PGC1-α was greater in 0P, and FGF21 and irisin were greater in 0P and 10P compared with 15P rats (Fig. 7a–e). Further, key regulatory transcripts in amino acid metabolism that were upregulated include solute carrier family 38 member 2 (SLC38A2) in 0P, SLC7A5 in 0P and 5P, and branched chain keto acid dehydrogenase E1, beta polypeptide (BCKDHB) in 0P and 10P (Fig. 7f–h).

## Discussion

We provide evidence that isocaloric diets with graded doses of protein produce divergent effects on energy intake, energy expenditure, plasma amino acids and gut hormones, and metabolic markers in peripheral tissues in obesity-prone rats. First, a protein-free diet (0P) decreased energy intake and increased energy expenditure, very low protein diet (5P) increased intake and expenditure, whereas moderately low protein diet (10P) increased intake with no change in expenditure. On realimentation to control diet (15P), 0P had transient anorexia but increased energy expenditure. Second, enhanced serotonergic and sympathetic signaling mediated the differential effects of low protein diets on energy balance. Blockade of 5HT3 receptors with ondansetron produced hyperphagia in 0P, 5P and 10P, but decreased energy expenditure in 10P, indicative of a role for higher serotonergic tone in inhibiting energy intake across these groups but in increasing energy expenditure in the 10P group. Propranolol (β-AR antagonist) decreased energy expenditure in 0P, 5P and 10P which, in part, is supportive of enhanced sympathetic drive in the metabolic adaptations to low protein diets. However, the increased energy expenditure is likely mediated through parallel or interdependent mechanisms because the mRNA abundance of key thermogenic markers in BAT (β2-AR, β3-AR, PGC1-α, UCP1, FGF21, β-Klotho, SERT, TPH1) and skeletal muscle (PGC1-α, FGF21, irisin) were increased with variable degrees of protein restriction. Third, 0P and 5P decreased plasma concentrations of multiple essential amino acids, 0P decreased anorexigenic hormones (PYY, leptin, insulin), but 0P and 5P increased the tissue expression and plasma concentrations of the metabolic hormone FGF21. Fourth, dietary protein deficiency produced disparate effects on body weight and composition. During protein restriction, body weight, fat and lean mass were decreased in 0P, body weight and lean mass were reduced in 5P, whereas these body compartments were unaltered in 10P. However, during the realimentation period, 10P gained weight and fat mass whereas the reduction in weight, fat and lean mass were sustained in the 0P and 5P groups. Further, the increased hepatic lipid content in 0P and 5P is supported by an increase in mRNA abundance of key lipogenic markers (CD36, FAS) and a reciprocal decrease in abundance of a lipolytic protein (HADH). Thus, these data demonstrate that dietary protein deficiency differentially modulates energy balance and metabolism.

The hypophagic effect of protein-free diets, and hyperphagic effect of very low protein and moderately low protein diets, in the current study are consistent with the ‘protein leverage’ hypothesis^2^, as well as previous studies on protein restriction in rats, mice and humans^1^,^6^,^7^,^8^,^9^,^14^,^17^,^22^. Although the low protein-induced hyperphagia is associated with a central orexigenic drive^11^,^15^,^16^,^17^,^18^, the peripheral signals that transmit information to central neural networks are poorly defined. To discern potential peripheral mediators, we focussed on circulating concentrations of amino acids, anorexigenic gut hormones, and peripheral serotonergic 5HT3, β-adrenergic and FGF21 signaling. Similar to other reports^16^,^17^, in the current study, protein restriction produced a dose-dependent decrease in majority of the essential amino acids. Interestingly, arginine, serine and glycine increased with concomitant anorexia in the 0P group. Protein deprivation decreased plasma concentrations of PYY and GIP, due in part to consumption of less food by animals in this group; hence, it is unlikely that these hormones mediate the anorexic effects of protein-free diets. Previous studies have shown that gut serotonergic signaling at peripheral 5HT3 receptor mediates the acute hypophagic effects of dietary carbohydrate in rats^29^,^30^,^31^. We extend these findings and demonstrate that ondansetron, a selective 5HT3 receptor antagonist, increased energy intake in rats that were fed isocaloric diets containing 0%, 5% and 10% protein with 67%, 62% and 57% carbohydrate calories, respectively. Therefore, as gut is a major source (~95%) of serotonin^25^, enhanced endogenous serotonin primarily of gut origin acting via 5HT3 receptors mediates the effects of diets low in protein, but comparatively high in carbohydrate, on energy intake.

There is limited evidence on the temporal changes in energy expenditure and substrate utilization with dietary protein deficiency. In the current study, the mean daily energy expenditure was greater in the 5P rats which was coincidental with an earlier hyperphagia during restriction. Despite an initial increase in energy expenditure, this effect did not persist in the 0P likely due to the sustained anorexia and weight loss. Previous studies have shown that an increased sympathetic influx to BAT is required for the thermogenic effects of low protein diets^19^,^20^,^21^,^22^,^23^. In the present study, using propranolol, a β-AR antagonist, we demonstrate that energy expenditure was dose-dependently attenuated in animals fed low protein diets with maximal attenuation in protein deprived rats. In support of enhanced sympathetic signaling, there was increased transcript abundance of β2-AR, β3-AR, PGC1-α and UCP1 in the BAT of low protein animals. Interestingly, majority of the BAT transcripts exhibited a non-linear parabolic response to the reduction in protein content with maximal responses between 10P and 5P and an attenuation with 0P. There are striking similarities between our findings and what others have shown with beta adrenergic signaling under cold acclimation. Chronic exposure to cold, and associated increase in sympathetic drive, has been shown to cause β-adrenergic receptor desensitization with a reduction in β3 transcripts in brown fat^39^,^40^,^41^,^42^. Because propranolol produced maximal attenuation of energy expenditure in 0P, it is likely that the greater and chronic sympathetic drive in the 0P lead to an adaptive attenuation of an increase in transcripts for β-adrenergic receptor and their downstream effectors PGC1α and UCP1. In addition, we also found a nearly dose-dependent upregulation of transcripts for FGF21 in the liver and muscle, increased plasma FGF21, and increased mRNA abundance for both FGF21 and its co-receptor β-Klotho in the BAT with low protein diets. Since the sympathetic system increases FGF21 expression and secretion from brown fat^43^, and FGF21 enhances sympathetic drive to brown fat^44^, the observed changes in FGF21 and β-klotho transcripts could likely be due to a reciprocal local or systemic feed-forward mechanism between FGF21 and sympathetic systems. Apart from adrenergic control, FGF21 expression and secretion could also be regulated by amino acids. Previously, dietary restriction of methionine^45^ or leucine^46^ has been shown to upregulate the tissue expression and circulating FGF21 concentrations in mice. We found greater plasma FGF21 concentrations in 0P and 5P rats which also had a concurrent decrease in plasma methionine and tyrosine but increase in glycine concentrations. In addition, the 10P had greater transcript abundance of FGF21 in brown fat and muscle together with a reduction in plasma threonine, tryptophan, valine, phenylalanine, leucine, isoleucine and lysine and an increase in serine and alanine. Whether alterations in these amino acids, either alone or together, contribute to the observed low protein diet-induced changes in FGF21 expression in multiple tissues remains to be determined. The relatively greater increase in FGF21 transcript in the liver than brown fat in 0P suggests that the source of FGF21 may shift from brown fat to the liver depending on the degree of protein restriction. Although liver-derived FGF21 may act through an endocrine mechanism to enhance BAT thermogenesis^17^,^47^, our data suggest that enhanced paracrine or autocrine FGF21 signaling in the BAT is also important for the effects of low protein diets on thermogenesis.

In addition to enhanced sympathetic drive, we provide evidence that increased serotonergic signaling acting via 5HT3 receptors is also important for the increased expenditure. Previously, it was shown that metergoline, a non-selective 5HT receptor antagonist and dopamine agonist, reduced resting VO_2_ in animals fed 8% protein diets^48^. In our study, the selective 5HT3 receptor antagonist ondansetron decreased energy expenditure particularly in the 10P group. Given that about 15% of circulating ondansetron gains access to the brain^49^, the effects of the antagonist on expenditure in the current study are likely mediated via both peripheral and central mechanisms. Although brain-derived serotonin may play a role in thermogenesis^28^, the increased transcript abundance of TPH1 and SERT in the BAT of 10P and 5P rats is suggestive of an upregulation of local serotonin turnover, which in turn might act via a paracrine or autocrine manner to enhance thermogenesis. Together, these findings suggest that increased adaptive thermogenesis of low protein diets is likely mediated through the convergence of parallel or interdependent sympathetic, serotonergic and FGF21 signaling pathways.

In the current study, reduction in body weight gain and tissue reserves in animals fed 0 to 5% protein diets is in agreement with previous studies^6^,^8^,^9^,^14^,^17^,^34^. As expected, the 0P with the lowest dietary ratios of protein to carbohydrate (0:67) or fat (0:33) had the lowest fat and lean tissue mass during restriction. The anorexia with consequent increase in lipid utilization, coupled with increased energy expenditure, likely contributed to a sustained reduction in weight gain, fat and lean mass in 0P. The lower ratio of protein to carbohydrate (5:62) in the 5P diet led to a reduction in lean mass without changes in fat mass, which contributed to an apparent increase in fat% during restriction. The reduction in weight and lean mass in 5P is likely due to increased energy expenditure that is not compensated for by the hyperphagia to maintain energy balance. Interestingly, the 5P animals appear to protect their fat mass while shifting their substrate oxidation from fats towards carbohydrates. The preferential oxidation of carbohydrates in 5P is reflective of the higher dietary carbohydrate content and is consistent with other reports^50^, and the partitioning of dietary fat towards adipose reserves is also similar to other studies with adipogenic effects of low protein-high carbohydrate diets in mice^51^. Previous studies have shown that following protein restriction, rats attempt to regain body weight and adipose reserves on refeeding with a standard protein diet^34^,^35^,^37^,^38^; however, the time-course of changes in tissue compartments and substrate utilization was relatively unknown. We demonstrate that during the realimentation period, 0P and 5P had decreased body weight, body fat and lean mass. Interestingly, on realimentation, 0P exhibited a robust preference for carbohydrate utilization, despite all groups being fed a common diet, and the greater lean% in 0P and 5P suggest that both groups rapidly partition dietary protein towards replenishing protein reserves. Importantly, 10P had increased body weight and body fat content on realimentation to a standard protein diet despite a lack of body composition differences during restriction. The greater feed efficiency of these animals during the realimentation resulted in increased weight gain with excess calories being partitioned towards adipose reserves. Thus, prior protein restriction exerts divergent long-term effects on body composition and substrate utilization with severe protein restriction delaying fat and lean accretion and enhancing carbohydrate use during realimentation, whereas moderate protein restriction predisposes to weight gain and obesity. These findings have important implications for long-term consequences of protein restriction on adiposity.

Despite lack of alterations in total body fat in 5P and 10P during protein restriction, we found that these animals, together with the 0P, had greater liver fat% indicative of hepatic lipidosis. In support of fat accumulation in the liver, we found that 0P rats had decreased protein abundance of the hepatic lipolytic marker HADH, with a reciprocal increase in the mRNA abundance of the key lipogenic markers CD36 and FAS. In our study, the low protein diets contained 67–57% carbohydrates with 33% fat. Others reported that diets relatively high in carbohydrate and fat, similar to the ranges in our study, promoted development of fatty liver and impaired glucose tolerance in both rats^52^,^53^,^54^ and mice^51^, whereas low protein-high carbohydrate diets increased adiposity and fatty liver with paradoxical improvement in glucose tolerance in mice^6^,^32^,^33^. Therefore, despite weight loss with 0P and 5P diets, the increased hepatic lipidosis likely negated any improvements in glucose tolerance. We next focused on key markers of amino acid metabolism in the liver and skeletal muscle. As expected, protein deprivation lead to an upregulation of the hepatic amino acid sensor GCN2 and its downstream targets peIF2α (Ser^51^):eIF2α and ATF4. We also observed distinct changes in key regulators of amino acid metabolism. The upregulation of transcripts for the amino acid transporters SLC7A5 and SLC3A2, and the rate-limiting enzyme in branched chain amino acid catabolism in the liver and muscle BCKDH, of particularly the 0P rats, is indicative of enhanced uptake and metabolism of branched chain amino acids by these tissues.

**Figure 8:**
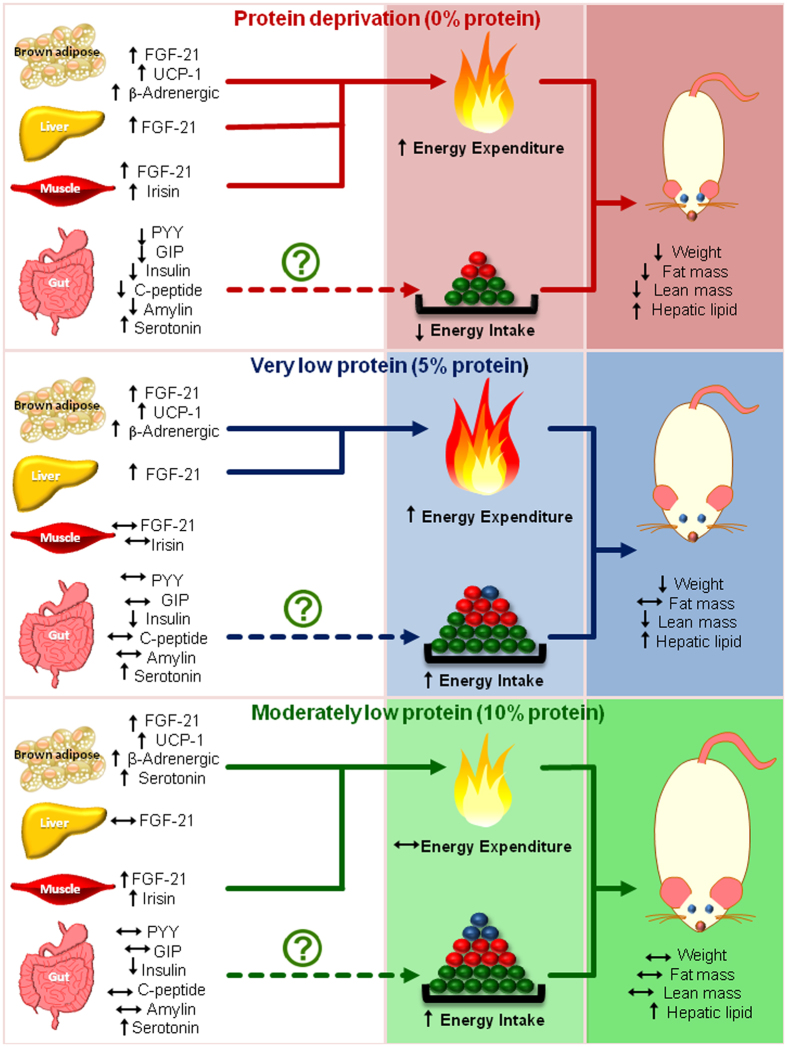
Model of potential mechanisms by which low protein diets modulate energy balance. Protein-free diets decrease energy intake and enhance energy expenditure resulting in loss of body weight, fat and lean mass, whereas very low protein diets promote hyperphagia and thermogenesis with resultant reduction in weight and lean mass, and moderately low protein diets are hyperphagic without altering energy expenditure and body fat and lean mass. Enhanced sympathetic, serotonergic and fibroblast growth factor-21 (FGF21) secretion and signalling likely contribute to the thermogenic effects of protein-free and very low protein diets. It is unlikely that anorexigenic gut peptides play a role in modulating intake, however, enhanced serotonergic signalling, likely of gut origin, mediates the effects of low protein diets on food intake. Further, dietary protein deficiency promotes hepatic lipidosis. Arrows pointing upwards, downwards or horizontally indicate an increase, decrease or no change, respectively. Pathways that need further validation are indicated by dashed lines. The colored circles represent relative proportions of protein (blue), carbohydrate (green) and fat (red) in the diet, respectively.

A potential caveat with our model is that the obesity prone OP-CD rats, which were originally developed from Sprague Dawley rats, have undergone multiple generations of breeding, and we did not test the effects of low protein diets in the control obesity-resistant strain. However, it is noteworthy that the hyperphagia, augmented thermogenesis and reduction in weight gain with our 0P and 5P, and the hyperphagia with 10P, is consistent with numerous other studies reporting similar findings in normal lean Sprague Dawley rats^7^,^8^,^9^,^19^,^21^,^55^,^56^. Although, the thermogenic activity of brown adipose tissue to dietary stimuli is often weak in obese animals^57^, the augmented thermogenesis with dietary protein restriction in our OP-CD rats suggests that effects of low protein diets on energy balance might be conserved in both lean and obese phenotypes. The potential mechanisms by which low protein diets modulate energy balance are depicted in Fig. 8. We provide evidence that severe protein deprivation produces a state of negative energy balance that persists beyond the period of deprivation primarily due to a decrease in energy intake and an increase in energy expenditure. In contrast, moderate protein deficiency produces hyperphagia without altering energy expenditure and predisposes to weight gain, adiposity and hepatic lipidosis. We also demonstrate that protein deficiency engages sympathetic and serotonergic signaling primarily in BAT to induce thermogenesis. Together, our findings demonstrate that dietary protein deficiency exerts divergent effects on multiple metabolic parameters in obesity-prone rats. Given that moderately low protein diets promote hyperphagia in humans^1^,^10^,^11^, our data, with an animal model that better represents human obesity, indicate that such diets could exacerbate pre-existing susceptibility to weight gain and obesity.

## Methods

### Animals, housing and treatments

The animal experiments were approved by the University of Calgary Animal Care Committee (#AC12–0033). Male obesity-prone Sprague Dawley rats (~155 g, 6 weeks old; Crl: OP-CD, Strain 463; Charles River, Montreal, QC, Canada) were selected as they capture the hallmarks of human obesity including polygenic inheritance, glucose intolerance and obesity^58^,^59^,^60^; hence, they would have a greater translational significance for testing the obesogenic effects of low protein diets. They were housed individually in metabolic cages of the Comprehensive Lab Animal Monitoring System (CLAMS^®^, Columbus Instruments; Columbus, OH, USA) under standard temperature (23–24 °C) and lighting conditions (12 hours light-dark cycle; lights off at 1100 h). The general maintenance and husbandry (Supplementary Methods) was according to our previously published procedures^5^.

Prior to testing, animals were acclimatized to the environment and experimental conditions for 2 weeks. During the acclimatization period, they received a standard chow diet (25% protein, 62% carbohydrate, 13% fat, energy density 4.07 kcal/g; PicoLab® Rodent Diet 20; LabDiet, St. Louis, MO, USA) for 4 days, followed by a high-fat control diet for 10 days (Supplementary Table S1). The rats (279 ± 3 g body weight) were then weight-matched and randomly allocated to four isocaloric high-fat diets (4.4 kcal/g; 33% fat calories) with protein contributing to 15% (control; 15P), 10% (10P), 5% (5P) or 0% (0P) calories. These diets represent arbitrary states of protein starvation or total deprivation (0P), very low (5P) and moderately low (10P) dietary protein, relative to recommended control (15P) requirements^61^. Diets were made in-house (Supplementary Table S1) using ingredients from Dyets, Inc. (Bethlehem, PA, USA). Three experiments were conducted. In experiment 1, to determine the metabolic responses to the duration of protein deprivation, rats (n = 8/group) were randomized to 0P for 14 days, 0P for 21 days, or 15P, during the restriction phase followed by a realimentation phase with feeding on 15P diet for 28 or 21 days. Since the metabolic responses were similar between 14 *vs* 21 days of protein deprivation, in experiment 2, rats (n = 4–8/group) were randomized to either 15P, 10P, 5P or 0P for 14 days of restriction followed by 14 days of realimentation on the 15P diet. Multiple energy balance parameters were measured in both phases. In experiment 3, the rats (n = 8–10/group) were randomly assigned to identical treatment groups as indicated above for experiment-2, and fed the test diets for 14 days followed by a meal challenge and subsequent tissue sampling.

### Metabolic measurements

Food intake and energy expenditure were recorded daily using CLAMS^®^ throughout the study (Supplementary Methods) as we reported previously^5^. The rats were weighed twice a week and body composition was measured weekly using a Minispec LF110® NMR Analyzer (Bruker Corporation, Milton, ON, Canada). IPGTT was performed in all animals at d 10–13 as we previously described^5^,^62^. Gross energy content (kilocalories per gram) of fecal samples collected towards the end of the first and second week of the study were analyzed by bomb calorimetry (1341 Plain Jacket Bomb Calorimeter, Parr Instrument Company, Moline, IL, USA) and energy digestibility calculated from the differences between total energy intake and fecal energy output.

### Blockade of 5HT3 receptors and β-AR

To assess the role of 5HT3 receptors and β-AR in energy balance, ondansetron (Ondansetron hydrochloride, Tocris, Burlington, ON, Canada, #2891) a selective 5HT3 receptor antagonist, and propranolol (Propranolol hydrochloride; Sigma-Aldrich, Oakville, ON, Canada, #P8688) a β1 and β2-AR blocker, were administered on d 6–8 and 15–17, respectively. In a cross-over design, following an overnight fast, each animal received intraperitoneal injection of saline or ondansetron (0.5 ml; 1 mg/kg in sterile 0.9% saline)^29^,^30^,^31^ at 1030 h (30 min before the onset of dark period) with ~48 h between injections. Similarly, overnight fasted rats received subcutaneous injection of either saline or propranolol (0.5 ml; 10 mg/kg in sterile 0.9% saline)^20^,^63^.

### Meal test and tissue harvesting

In experiment 3, on d 19, following an overnight fast, rats were allowed to freely consume their usual treatment diet for 1 h after dark onset (1100 h). Blood samples were obtained from the saphenous vein before (0 min) and at 60 and 120 min after onset of food access, plasma separated, and various tissues sampled at termination (Supplementary Materials and Methods). Blood glucose concentrations were measured using a glucometer at the above mentioned time points (Accu-Chek®; Roche Diagnostics, QC, Canada).

### Plasma hormones and amino acids

Plasma concentrations of PYY, GIP, amylin, insulin, C-peptide and leptin were measured in duplicate using a Milliplex® Map rat gut hormone panel (Millipore, Luminex Corp., Austin, TX; RGT 88 K) on a Luminex® platform (Bio-Plex 200) following our published procedures^5^,^62^. Plasma FGF21 concentrations were measured using a commercially available rat/mouse FGF21 ELISA kit (EMD Millipore Corporation, Saint Charles, MO, USA, #EZRMFGF21–26 K). The intra-assay coefficient of variation for PYY, GIP, amylin, insulin, C-peptide, leptin and FGF21 were 8.42, 19.21, 11.45, 3.46, 5.03, 8.13 and 3.75%, respectively. Terminal postprandial samples were used for measuring plasma amino acid concentrations (Supplementary Materials and Methods).

### Immunoblot and reverse transcription semi-quantitative real-time polymerase chain reaction (RT-qPCR) analyses

Immunoblotting was performed (see Supplementary Methods and Supplementary Table S5) for eIF2α, peIF2α (Ser^51^), ATF4, and HADH in liver following our published procedures^5^,^62^,^64^. RT-qPCR was performed (see Supplementary Methods and Supplementary Table S6) for GCN2, ATF4, FGF21, β-Klotho, SLC38A2, SLC7A5, SLC3A2, BCKDH, CD36, FAS, CPT1, SERT, TPH1, β3-AR, UCP1, UCP3, irisin, and PGC1-α in BAT, muscle and liver following our published procedures^5^,^62^,^64^.

### Statistical analysis

Repeated measures on energy intake, energy expenditure, body composition, body weight, IPGTT and plasma hormones were analyzed by linear mixed models using SPSS (IBM® SPSS® Statistics Version 22, Armonk, NY, USA). Metabolic measurements during the 14-day protein restriction phase of experiments 1 to 3 were combined prior to analyses. The fixed effects of dietary treatment, time and the interaction of dietary treatment and time were included in the model. In addition, energy expenditure was also analyzed by incorporating lean mass as a covariate in the above model, followed by ANCOVA at each time point. For ondansetron and propranolol effects on energy intake and energy expenditure, data were modeled to include fixed effects of dietary treatment, drug, time and interactions of dietary treatment, drug and time. Animal nested in dietary treatment was the random variable on which repeated measures were taken and covariance structures modeled either as compound symmetry, heterogenous compound symmetry, first-order antedependence, autoregressive, heterogenous autoregressive or toeplitz. Discrete data on plasma amino acids, AUC for drug effects on energy expenditure, digestible energy, feed efficiency, and protein and mRNA abundance of tissue markers, were analyzed by one-way ANOVA with dietary treatment as a between-subject factor. Means were separated by Dunnett’s post hoc test with 15P dietary treatment as the control. For drug effects within groups, paired t-test was used to separate means. Data are presented as the mean ± standard error of the mean (SEM). *P* values < 0.05 were considered to declare significant difference and trends were indicated at *P* values < 0.10.

## Additional Information

**How to cite this article**: Pezeshki, A. *et al.* Low protein diets produce divergent effects on energy balance. *Sci. Rep.* **6**, 25145; doi: 10.1038/srep25145 (2016).

## Supplementary Material

Supplementary Information
