# Supplementary material for: Low protein diets produce divergent effects on energy balance
Source: Sci Rep. 2016 Apr 28;6:25145. doi: 10.1038/srep25145 (PMC4848496; doi:10.1038/srep25145)
Supplement: Supplementary Information [file srep25145-s1.pdf]

Supplementary Information

Low Protein Diets Produce Divergent Effects on Energy Balance

Adel Pezeshki,[1,2a] Rizaldy C. Zapata,[1] Arashdeep Singh,[1] Nicholas J. Yee,[1] and Prasanth K. Chelikani[1,2,*]

[1]Department of Production Animal Health, Faculty of Veterinary Medicine; [2]Gastrointestinal Research Group, Snyder Institute for Chronic Diseases; University of Calgary, Calgary, Alberta, Canada, T2N 4N1.

*Corresponding author:

Prasanth K. Chelikani, PhD

Department of Production Animal Health, Faculty of Veterinary Medicine

Gastrointestinal Research Group, Snyder Institute for Chronic Diseases

University of Calgary, 3330 Hospital Drive NW

Calgary, Canada, T2N 4N1.

Phone: (403) 210-7652

Fax: (403) 210-6693

E-mail: pchelika@ucalgary.ca

[a] Current address: Department of Animal Science, Oklahoma State University, Stillwater, OK 74078, USA

Supplementary Information:

Supplementary Figures:

Figure S1. Effects of protein-free diets on energy balance.

Figure S2. Effects of low protein diets on energy balance.

Figure S3. Effects of low protein diets on energy intake.

Figure S4. Effects of low protein diets on energy expenditure.

Figure S5. Effects of low protein diets on glucose tolerance.

Supplementary Tables:

Table S1. Diet composition.

Table S2. Effects of low protein diets on organ weights and energy digestibility.

Table S3. Plasma concentrations (nmol/mL) of essential and nonessential amino acids in obesity-prone rats after 14 days of protein restriction.

Table S4. Feed efficiency during protein restriction and realimentation periods in obesity-prone rats.

Table S5. The host, dilution and supplier of primary and secondary antibodies for immunoblotting.

Table S6. The primer sequence (forward, F, and reverse, R), location on template (base pairs, bp), amplicon size, and GenBank accession numbers for target and reference genes used for qPCR in the current study.

Supplementary Materials and Methods:

General Maintenance and Husbandry.

Measurements of Food Intake and Energy Expenditure.

Blood Sampling and Tissue Harvesting.

Measurement of Plasma Amino Acid Concentrations.

Immunoblot Analyses.

RNA Isolation and Reverse Transcription Semi-Quantitative Real-Time Polymerase Chain Reaction (RT-qPCR)

Supplementary References:

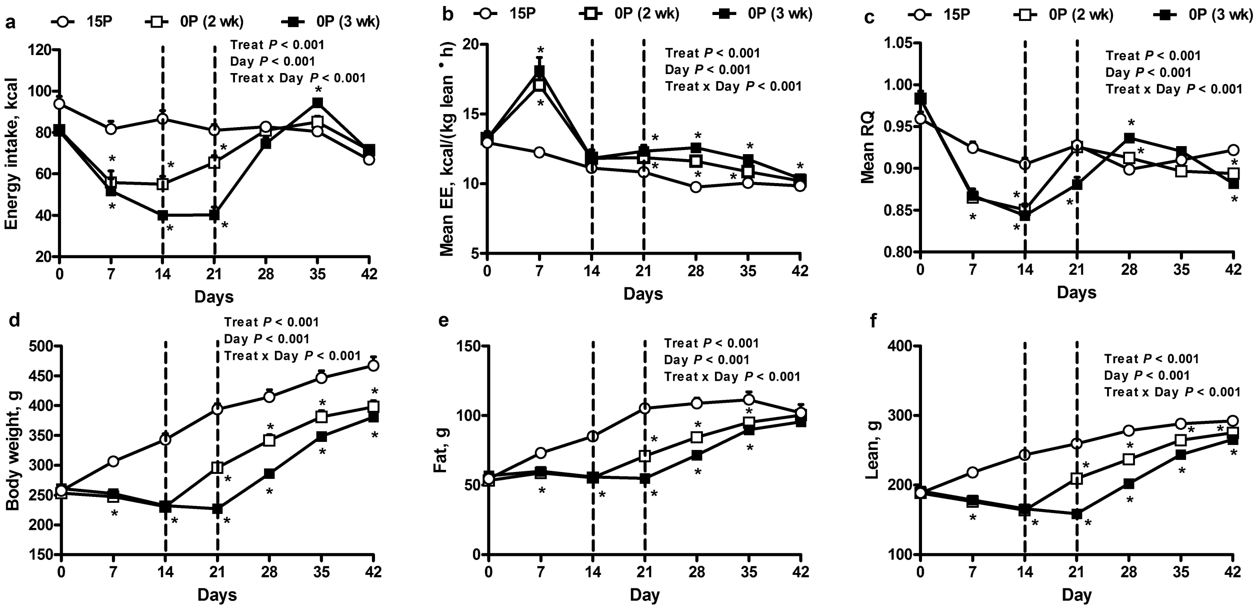

Supplementary Figure S1. Effects of protein-free diets on energy balance. (a) Daily energy intake, (b) mean energy expenditure (EE) in the dark period, (c) mean respiratory quotient (RQ) in the dark period, (d) body weight, (e) body fat mass, and (f) body lean mass of obesity-prone rats. Following feeding isocaloric diets including either a control (15% protein; 15P) or protein-free (0% protein; 0P) diets for 14 and 21 days, all animals were provided the control diet (15P) during a realimentation phase. Dotted lines separate the restriction and realimentation phases. Values are mean ± SEM, n=8/group. *$P < 0.05$ *vs* 0P.

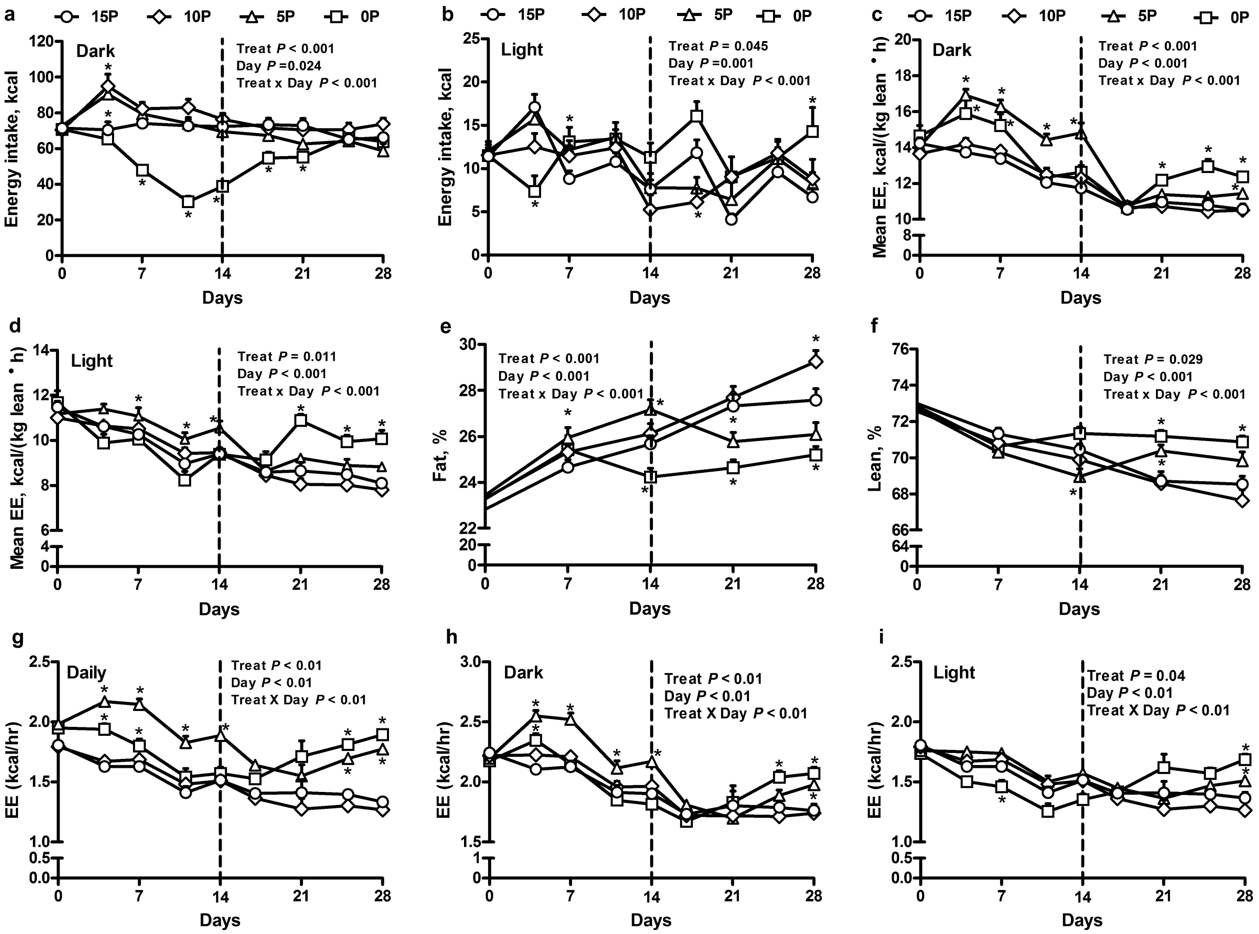

Supplementary Figure S2. Effects of low protein diets on energy balance. (a) Dark period energy intake, (b) light period energy intake, (c) mean energy expenditure (EE) in the dark period, (d) mean energy expenditure in the light period, (e) body fat percentage, (f) body lean percentage, (g) total daily heat production using lean mass as covariate, (h) dark period heat production using lean mass as covariate and (i) light period heat production using lean mass as covariate in obesity-prone rats. Following feeding different dietary treatments for 14 days, all animals were provided the control diet (15% protein) for 14 days during a realimentation phase. Dotted line separates the restriction and realimentation phases. Values are mean ± SEM, n=13-16/group. *$P < 0.05$ *vs* 15P, 15% protein; 10P, 10% protein; 5P, 5% protein; 0P, 0% protein.

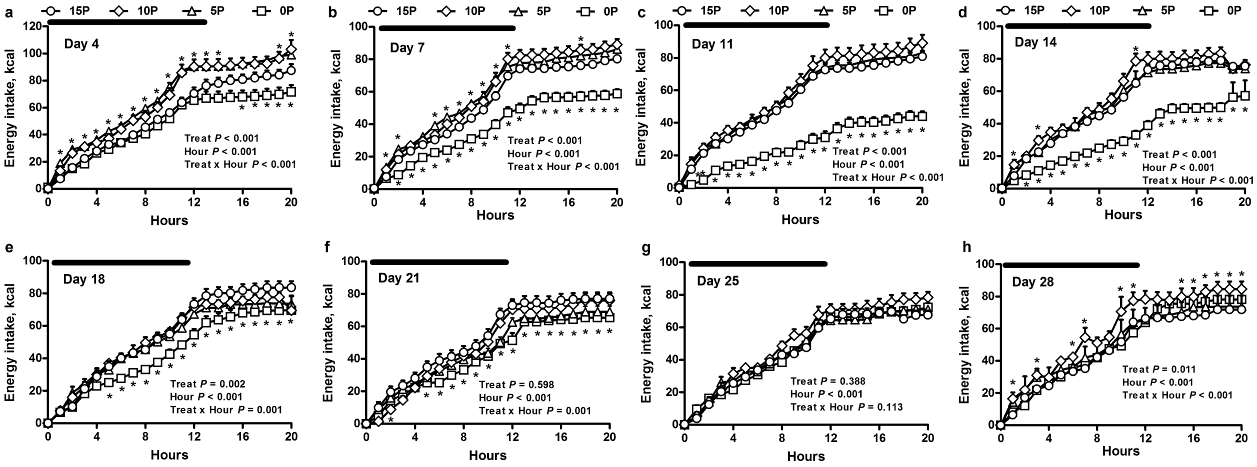

Supplementary Figure S3. Effects of low protein diets on energy intake. Cumulative hourly caloric intakes for (a) day 4, (b) day 7, (c) day 11, (d) day 14, (e) day 18, (f) day 21, (g) day 25, and (h) day 28 in obesity-prone rats. Following feeding different dietary treatments for 14 days, all animals were provided the control diet (15% protein) for 14 days during a realimentation phase. Black bar denotes the dark period. Values are mean ± SEM, n=13-16/group. **P* < 0.05 *vs* 15P, 15% protein; 10P, 10% protein; 5P, 5% protein; 0P, 0% protein.

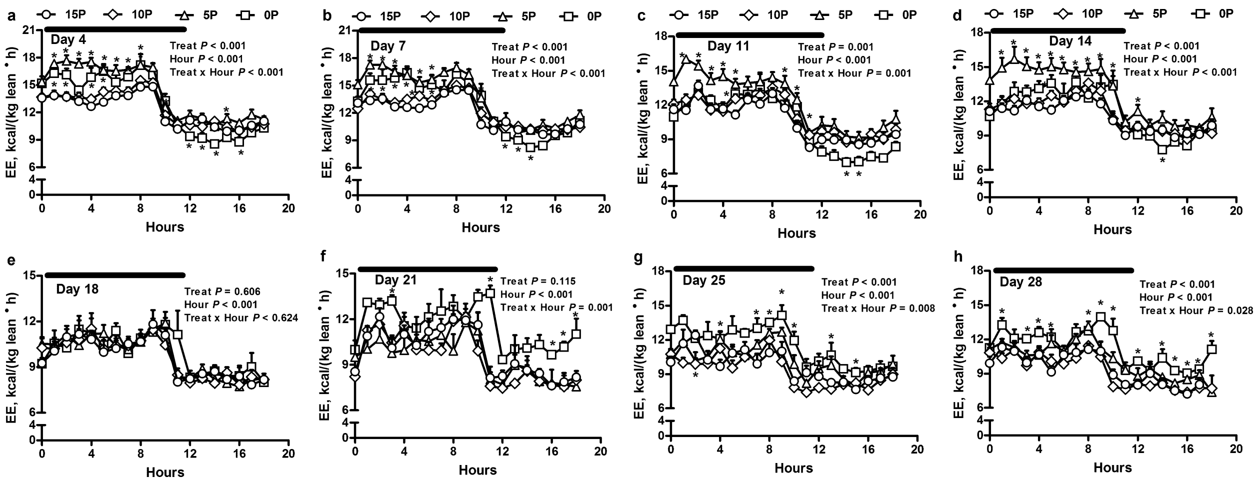

Supplementary Figure S4. Effects of low protein diets on energy expenditure. Changes in hourly energy expenditure (EE) for (a) day 4, (b) day 7, (c) day 11, (d) day 14, (e) day 18, (f) day 21, (g) day 25, and (h) day 28 in obesity-prone rats. Following feeding different dietary treatments for 14 days, all animals were provided the control diet (15% protein) for 14 days during a realimentation phase. Black bar denotes the dark period. Values are mean ± SEM, n=13-16/group. **P* < 0.05 *vs* 15P, 15% protein; 10P, 10% protein; 5P, 5% protein; 0P, 0% protein.

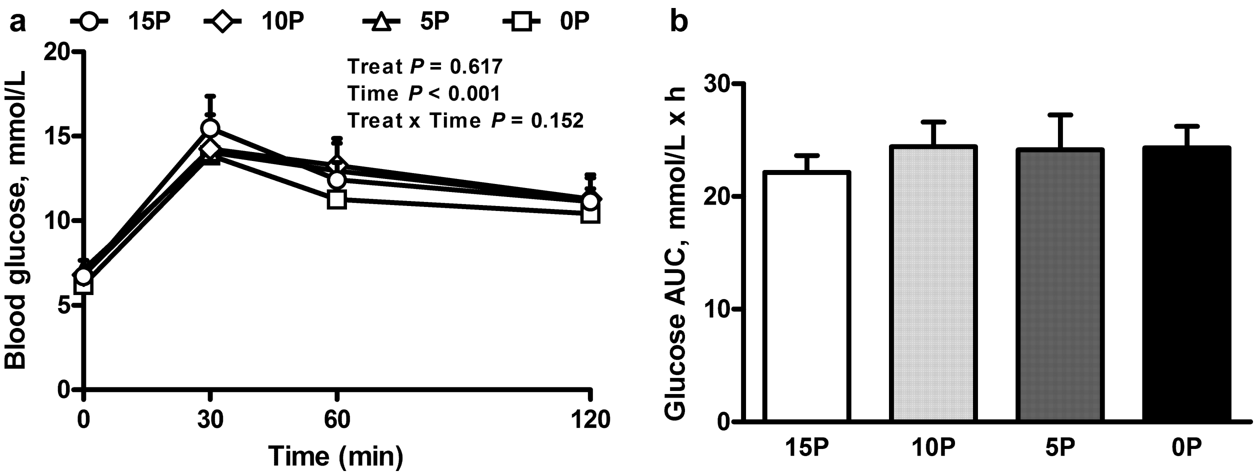

Supplementary Figure S5. Effects of low protein diets on glucose tolerance. (a) Blood glucose concentrations, and (b) blood glucose area under the curve (AUC), following an intraperitoneal glucose tolerance test in obesity-prone rats after 10 to 13 days of protein restriction. Values are mean ± SEM, n=7/group. 15P, 15% protein; 10P, 10% protein; 5P, 5% protein; 0P, 0% protein.

Supplementary Table S1. Diet composition.

**Ingredients**	**15% protein (15P)**	**10%protein (10P)**	**5%protein (5P)**	**0%protein (0P)**
Corn starch [1] (g)	476	531	586	641
Albumin (egg white) [1] (g)	165	110	55	0
Sucrose [1] (g)	100	100	100	100
Corn oil [1] (g)	60	60	60	60
α-Cellulose [1] (g)	50	50	50	50
AIN-93-MX [1] (g)	35	35	35	35
AIN-93-VX [1] (g)	10	10	10	10
L-Cystine [1] (g)	1.8	1.8	1.8	1.8
Choline bitartrate [1] (g)	2.5	2.5	2.5	2.5
Lard [1] (g)	100	100	100	100
Total amount (g)	1000	1000	1000	1000
Total protein content (% kcal)	15%	10%	5%	0%
Total carbohydrate content (% kcal)	52%	57%	62%	67%
Total fat content (% kcal)	33%	33%	33%	33%
Energy density (kcal/g) [2]	4.40	4.40	4.40	4.40

[1] Diets were prepared in-house using ingredients from Dyets Inc. (Bethlehem, PA, USA).
[2] Energy density is calculated from the calorifc values of protein, fat and carbohydrate at 4, 9 and 4 kcal/gm respectively.

Supplementary Table S2. Effects of low protein diets on organ weights and energy digestibility. Relative weights (as a percentage of body weight) of heart, kidney and liver, liver fat% as measured by NMR, and energy digestibility (kcal/day) by bomb calorimetry in obesity-prone rats. Values are mean ± SEM, n=8/group.

	**15% protein (15P)**	**10% protein (10P)**	**5% protein (5P)**	**0% protein (0P)**
Heart (%)	0.32 ± 0.01	0.31 ± 0.01	0.32 ± 0.01	0.40 ± 0.03*
Kidney (%)	0.71 ± 0.02	0.70 ± 0.03	0.68 ± 0.03	0.74 ± 0.02
Liver (%)	3.22 ± 0.08	3.38 ± 0.07	3.72 ± 0.13*	3.29 ± 0.13
Liver fat%	14.1 ± 0.49	16.3 ± 0.57*	22.3 ± 1.09*	16.2 ± 1.07
Digestible energy (kcal/day; day 7)	74.3 ± 1.37	86.3 ± 4.67*	70.1 ± 4.23	43.5 ± 4.22*
Digestible energy (kcal/day; day 14)	78.7 ± 4.62	73.6 ± 2.12	69.2 ± 3.99	46.0 ± 2.61*

*$P < 0.05$ 15P *vs* 10P, 5P, 0P.

Supplementary Table S3. Plasma concentrations (nmol/mL) of essential and nonessential amino acids in obesity-prone rats after 14 days of protein restriction. Values are mean ± SEM, n=8/group.

**Plasma amino acids (nmol/mL)**	**15%protein (15P)**	**10% protein (10P)**	**5% protein (5P)**	**0% protein (0P)**
**Essential**				
Histidine	61.99 ± 5.046	43.12 ± 2.120*	46.61 ± 5.369*	75.19 ± 3.845
Threonine	223.6 ± 12.77	161. 1 ± 6.444*	111.8 ± 11.97*	82.03 ± 4.144*
Arginine	68.23 ± 15.80	51.95 ± 8.264	82.01 ± 14.62	123.9 ± 18.25*
Taurine	520.1 ± 83.97	401.9 ± 36.25	450.7 ± 54.79	452.1 ± 17.94
Tryptophan	95.23 ± 3.006	75.48 ± 2.466*	65.29 ± 7.273*	47.53 ± 5.080*
Methionine	68.73 ± 1.852	66.70 ± 2.070	49.43 ± 3.611*	32.34 ± 2.037*
Valine	225.1 ± 11.25	177.2 ± 5.675*	135.8 ± 6.226*	79.61 ± 4.912*
Phenylalanine	95.96 ± 3.849	82.59 ± 2.122*	70.18 ± 3.009*	42.44 ± 1.969*
Isoleucine	105.4 ± 5.938	86.66 ± 4.165*	63.67 ± 3.860*	31.75 ± 2.921*
Leucine	145.8 ± 9.444	105.5 ± 4.101*	88.02 ± 8.683*	58.66 ± 6.699*
Lysine	324.2 ± 13.26	275.6 ± 13.65*	269.1 ± 19.27*	196.4 ± 18.24*
**Non-Essential**				
Aspartic acid	28.74 ± 3.029	25.58 ± 2.65	32.78 ± 3.779	42.30 ± 10.11
Glutamic acid	180.2 ± 28.80	107.0 ± 38.03	218.8 ± 34.68	260.2 ± 29.43
Asparagine	74.09 ± 1.206	91.90 ± 23.88	64.21 ± 2.391	52.26 ± 2.049
Serine	189.9 ± 9.705	265.2 ± 16.25*	310.7 ± 11.18*	443.3 ± 33.77*
Glutamine	654.5 ± 35.20	648.4 ± 85.68	758.1 ± 35.19	820.7 ± 77.35
Glycine	300.7 ± 25.73	329.5 ± 33.48	406.2 ± 21.33*	568.9 ± 53.70*
Citrulline	87.64 ± 5.300	81.45 ± 9.958	88.02 ± 3.176	70.96 ± 6.162

Alanine	618.2 ± 40.04	861.3 ± 42.94*	950.8 ± 33.77*	804.1 ± 67.51*
Tyrosine	79.28 ± 2.269	78.06 ± 2.458	67.61 ± 4.620*	37.20 ± 1.801*
Ornithine	143.3 ± 11.47	141.1 ± 9.855	119.5 ± 10.24	85.84 ± 17.48*

*$P < 0.05$ 15P *vs* 10P, 5P, 0P.

Supplementary Table S4. Feed efficiency during protein restriction and realimentation periods in obesity-prone rats. Values are mean ± SEM, n=7-8/group.

**Feed efficiency (feed-to-gain ratio)** [1]	**15% protein (15P)**	**10% protein (10P)**	**5% protein (5P)**	**0% protein (0P)**
Week 1 (d 1-7)	12.51 ± 0.69	14.24 ± 0.74	28.39 ± 1.37	27.99 ± 46.99
Week 2 (d 8-14)	17.04 ± 0.90	19.31 ± 2.46	40.31 ± 3.99*	-17.20 ± 2.20*
Week 3 (d 15-21)	14.31 ± 0.69	14.97 ± 1.71	17.90 ± 1.56	-47.92 ± 26.13*
Week 4 (d 22-28)	21.61 ± 0.65	18.36 ± 0.85*	13.03 ± 0.57*	10.13 ± 0.54*

[1] Calculated as cumulative energy intake (kcal) / weight gain (g).
**P* < 0.05 15P *vs* 10P, 5P, 0P.

Supplementary Table S5. The host, dilution and supplier of primary and secondary antibodies for immunoblotting.

Name	Host	Dilution	Vendor
Anti-eIF2α [1]	Rabbit	1:1000	Abcam, Cambridge, MA, #ab97817
Anti-peIF2α (Ser[51])[1]	Rabbit	1:1000	EMD Millipore, Etobicoke, ON, #07-760
Anti-ATF4[1]	Mouse	1:500	Abcam, Cambridge, MA, #ab50546
Anti-HADH[1]	Goat	1:500	Santa Cruz Biotechnologies, Santa Cruz, CA, #SC74650
Anti-β-Actin	Mouse	1:500	Santa Cruz Biotechnologies, Santa Cruz, CA, #SC-47778
Anti-GAPDH[1]	Rabbit	1:500	Santa Cruz Biotechnologies, Santa Cruz, CA, #SC-25778
Anti-rabbit IgG[1]	Donkey	1:4000	GE Healthcare, Mississauga, ON, #NA934V
Anti-goat IgG[1]	Donkey	1:5000	Santa Cruz Biotechnologies, Santa Cruz, CA, #SC-2020
Anti-mouse IgG[1]	Goat	1:5000	Santa Cruz Biotechnologies, Santa Cruz, CA, #SC-2005

[1]eIF2α, eukaryotic initiation factor 2α; peIF2α (Ser[51]), serine 51 phosphorylated eukaryotic initiation factor 2α; ATF4, activating transcription factor 4; HADH, 3-hydroxyacyl-CoA dehydrogenase; GAPDH, glyceraldehyde 3-phosphate dehydrogenase; IgG, immunoglobulin.

Supplementary Table S6. The primer sequence (forward, F, and reverse, R), location on template (base pairs, bp), amplicon size, and GenBank accession numbers for target and reference genes used for qPCR in the current study.

Genes	Sequence (5′→3′)	Location on template (bp)	Amplicon size (bp)	GenBank accession no.
β3-AR[1]	**F** TAGCAAGGAGCCTGACTTCTG<br>**R** TTGGTTCTGGAGAGTTGCGG	**F** 1439-1459<br>**R** 1575-1556	137	NM_013108.2
TPH1[1]	**F** CCAGTGGCTTTGAGGTCCTCTTT<br>**R** CCCCTTTCTGAGGAATGGTCTT	**F** 52-74<br>**R** 167-146	116	NM_001100634.2
UCP1[1]	**F** CCGAGCCAAGATGGTGAGTT<br>**R** CCTTGGATCTGAAGGCGGAC	**F** 172-191<br>**R** 315-296	144	NM_012682.2
UCP3[1]	**F** AAAGGAACGGACCACTCCAG<br>**R** CTTCACCACATCCGTGGGTT	**F** 444-463<br>**R** 542-523	99	NM_013167.2
FNDC5 (Irisin)[1]	**F** GCCGAGAAGATGGCCTCTAA<br>**R** GCTATGACACCTGCCCACAT	**F** 386-405<br>**R** 513-494	128	NM_001270981.1
FGF21[1]	**F** CCTGGAGCTCAAAGCCTTGA<br>**R** AAACTGCAGGCCTCAGGATC	**F** 243-262<br>**R** 371-352	129	NM_130752.1
β-Klotho[1]	**F** CTCAAGAAGCCGACACATGC<br>**R** GGGTCATGAGAGCCACACAA	**F** 21-40<br>**R** 94-75	74	XM_008770179.1
β2-AR[1]	**F** GTGGATTGTGTCGGGCCTTA<br>**R** GCGATAGCATAGGCCTGGTT	**F** 670-689<br>**R** 804-785	135	NM_012492.2
SLC7A5[1]	**F** CAATCTGGACGTGGGGAACA<br>**R** CCAGGGGGAGGTTCCTGTAG	**F** 786-805<br>**R** 907-888	122	NM_017353.1
SLC3A2[1]	**F** CCCGAACCTACTGAACACTCC<br>**R** ATGGTACCTGAGTCGCCAGC	**F** 120-140<br>**R** 232-213	113	NM_019283.3
BCKDHA[1]	**F** GGGCTTGGCTAGATTCCACC<br>**R** GGGGATCTTCACTGGGGTTG	**F** 94-113<br>**R** 290-271	197	NM_012782.1
BCKDHB[1]	**F** TGCAAGGCTTCTTGCAACC<br>**R** GAGTTTGCCCATACTGAAGGG	**F** 123-141<br>**R** 227-207	105	NM_019267.1
SLC38A2[1]	**F** CGGCGACTATCTGGTCCTTC<br>**R** TCTTGCAAATCACCACAATCAGA	**F** 973-992<br>**R** 1112-1090	140	NM_181090.2
eIF2αK4 (GCN2)[1]	**F** CTCCGGAGAATCACCGCTAC<br>**R** TCCACCTGCACGTACACTTC	**F** 40-59<br>**R** 313-294	274	NM_001105744.2
ATF4[1]	**F** ATGGATGGGTTGGTCAGTGC<br>**R** CAACGTGGCCAAAAGCTCAT	**F** 460-479<br>**R** 618-599	159	NM_024403.2

CD36[1]	**F** TGCATGAATTAGTTGAACCAGGCCA<br>**R** CCACAGTTCCGATCGCAGCCC	**F** 130-154<br>**R** 256-236	127	NM_031561.2
FAS[1]	**F** GGACATGGTCACAGACGATGAC<br>**R** CGTCGAACTTGGACAGATCCTT	**F** 171-192<br>**R** 265-244	95	NM_017332.1
PGC1-α[1]	**F** GTGCAGCCAAGACTCTGTATGG<br>**R** GTCCAGGTCATTCACATCAAGTTC	**F** 56-77<br>**R** 176-153	121	NM_031347.1
SLC6A4[1]	**F** AGACAGGGGTGTGGGTAGAT<br>**R** TGACGAAGCCAGAGACGAAG	**F** 1072-1091<br>**R** 1242-1223	171	NM_013034.4
β-Actin	**F** GGATCAGCAAGCAGGAGTACGA<br>**R** AACGCAGCTCAGTAACAGTCCG	**F** 1145-1166<br>**R** 1229-1208	85	NM_031144.3

[1]β3-AR, β3-adrenergic receptors; TPH1, tryptophan hydroxylase 1; UCP1, uncoupling protein-1; UCP3, uncoupling protein-3; FNDC5, fibronectin type III domain-containing protein 5; FGF21, fibroblast growth factor 21; β2-AR, β2-adrenergic receptors; SLC7A5, solute carrier family 7 member 5; SLC3A2, solute carrier family 3 member 2; BCKDHA, branched chain keto acid dehydrogenase E1, alpha polypeptide; BCKDHB, branched chain keto acid dehydrogenase E1, beta polypeptide; SLC38A2, solute carrier family 38 member 2; eIF2αK4, eukaryotic translation initiation factor 2 alpha kinase 4 (GCN2, general control non-depressible 2); ATF4, activating transcription factor 4; CD36, cluster of differentiation 36; FAS, fatty acid synthase; PGC1-α, peroxisome proliferator-activated receptor gamma coactivator 1 α; SLC6A4, solute carrier family 6, member 4

Supplementary Materials and Methods

General Maintenance and Husbandry

The general maintenance and husbandry was according to our previously published procedures [1]. Briefly, the Comprehensive Lab Animal Monitoring System (CLAMS®, Columbus Instruments; Columbus, OH, USA) was started at 1100 h every day and stopped at 0900 h in the following day (22h measurement). General maintenance and husbandry including calibration of the sensors, filling of the feeders and water bottles were carried out between 0900 to 1100 h. Further, during this period, body weight and body composition measurements, intraperitoneal glucose tolerance test (IPGTT) and drug injections were conducted. Throughout the study period, the animals had *ad libitum* access to fresh food provided on alternate days and water except the days before IPGTT, drug injections and meal test when food was taken away but not water for 16 h (overnight fasting).

Measurements of Food Intake and Energy Expenditure

Food intake and energy expenditure were recorded daily by the CLAMS® system throughout the study as we reported previously [1]. Briefly, the rats had access to powdered food through a center feeder assembly resting on a balance, and the balance weights were recorded at ~30 sec intervals. The energy intake of individual rats was measured by multiplying the food intake (g) by physiological fuel value of diets (4.4 kcal/g). The total energy expenditure was measured by open circuit indirect calorimetry with negative flow controllers providing fresh-air to all chambers at 2 L/min. Air was sampled from each cage for 5 sec following a 55 sec stabilization period, and after every two cages, a reference air measurement was recorded. Each day before starting the CLAMS®, the O2 and CO2 sensors were calibrated with a calibration gas (20.50% O2, 0.5% CO2) at 5-10 psi. Oxygen consumption rate (VO2 ml/kg body weight/h), carbon dioxide production rate (VCO2 ml/kg body weight/h) and respiratory exchange ratio (RER) were measured and the total energy expenditure was computed as calorific value (CV) × VO2, where CV = 3.815 + 1.232 × RER [2] and data were expressed as kcal/(kg lean mass/h).

Blood Sampling and Tissue Harvesting

Following our previous procedures [1,3], blood samples were collected on ice into tubes containing ethylenediaminetetraacetic acid (EDTA; 1.5 mg/ml blood), protease inhibitor cocktail (10 µl/ml blood; Sigma-Aldrich, Oakville, ON, Canada) and dipeptidyl peptidase IV inhibitor (DPP-IV inhibitor; 10 µl/ml blood; Millipore Corporation, Temecula, CA, USA). Samples were immediately centrifuged at 4°C (4,000 rpm for 15 min), plasma was collected and stored at -80°C until further analyses. Following the last blood sampling, the animals were euthanized by using sodium pentobarbital (120 mg/kg IP; Euthanyl®, Bimeda-MTC, ON, Canada) and interscapular brown adipose (BAT), liver, leg skeletal muscle, kidney and heart tissues were collected, rinsed in sterile phosphate buffer saline, weighed and immediately snap-frozen in liquid nitrogen, and stored at -80°C until further analyses. Liver lipid content was measured using a biopsy probe of the Minispec LF110® NMR Analyzer (Bruker Corporation, Milton, ON, Canada).

Measurement of Plasma Amino Acid Concentrations

Plasma amino acid concentrations were measured as described previously [4]. Briefly, plasma amino acid concentrations were measured for terminal samples collected at 120 min (except

cystine, proline and hydroxy proline), by using a fluorometric high performance liquid chromatography method involving pre-column separation of amino acid derivatives using a derivatizing agent, *o*-phthaldialdehyde. Amino acids were separated by gradient elution from a Supelcosil LC-18 column (15 cm ×4.6 mm, 3 μm) and quantified with amino acid standards (Sigma-Aldrich, Oakville, ON, Canada).

Immunoblot Analyses

Immunoblotting was performed according to our published procedures [1,3,5]. Briefly, frozen liver samples were homogenized, sonicated, and supernatants collected, followed by quantification of protein concentration by Bradford assay (Biorad, Mississauga, ON, Canada). Protein extract (25 μg) from each animal was denatured at 95°C for 3 min and fractioned using 10% sodium dodecyl sulfate polyacrylamide gel electrophoresis (SDS-PAGE). The separated proteins were transferred to nitrocellulose membranes, and then blocked with 5% (m/v) skim milk or 5% (m/v) bovine serum albumin (BSA) in Tris-buffered saline containing 0.1% Tween-20 (TBST-5% milk or TBST-5% BSA). The membranes were incubated overnight at 4°C with either TBST-5% milk or TBST-5% BSA with various primary antibodies (Table S4). Subsequently, blots were incubated with Horseradish peroxidase-conjugated secondary antibodies (Table S4) in TBST-5% milk for 1 h at room temperature. Protein bands were captured using a ChemiDoc MP® imaging system (Bio-Rad Laboratories Ltd., Mississauga, ON, Canada). The band intensity of each target protein was normalized to the internal loading controls GAPDH or β-Actin (Table S4) for assessing the relative amount of protein content within the sample.

RNA Isolation and Reverse Transcription Semi-Quantitative Real-Time Polymerase Chain Reaction (RT-qPCR)

RNA isolation and RT-qPCR were performed following our published procedures [3,5]. Briefly, total RNA was isolated from iBAT, muscle and liver tissues (80-100 mg) according to the manufacturer's instruction (RNeasy Mini Kit; Qiagen Inc., Toronto, ON, Canada). Total RNA was treated with Deoxyribonuclease I, Amplification Grade (Invitrogen, Burlington, ON, Canada) according to the instruction of manufacturer to eliminate DNA. Complementary DNA (cDNA) was synthesized using 1.25 μg of RNA on a Mastercycler pro thermocycler (Eppendorf Canada Ltd., Mississauga, ON, Canada). The total reaction volume for cDNA synthesis was 20 μl containing: 5 μL of DNase-treated total RNA, 2 μL of random primers, 2 μL of deoxyribonucleotide triphosphate (dNTP) mix, 4 μL of 5× first-strand buffer, 2 μL of dithiothreitol (0.1 M), 1 μL of RNaseOUT (40 U/μL), 1 μL of Superscript II reverse transcriptase (200 U/μL), and 3 μL of RNase/DNase-free water (OmniPur; EMD Chemicals Inc., Mississauga, ON, Canada). The following program was used for cDNA synthesis: 22°C for 5 min, followed by 42°C for 30 min, 85°C for 5 min, and terminated at 4°C. All the reagents for cDNA synthesis were purchased from Invitrogen (Burlington, ON, Canada). qPCR analysis was performed in duplicate in a 96-well plate on a Mastercycler ep realplex thermocycler (Eppendorf Canada Ltd.). Each well (25 μl total volume) contained 12.5 μl of SYBR Green master mix (Applied Biosystems Inc., Foster City, CA, USA), 2 μL of cDNA, 0.2 μL of each primer (100 mM), and 10.1 μL of RNase/DNase-free water. Primers for target genes and housekeeping gene (β-actin) were designed using the Primer Express version 3.0 (Applied Biosystems Inc., Foster City, CA, USA) and their sequence is given in Table S5. qPCR conditions were as follows: denaturation: 50°C for 2 min and 95°C for 10 min, 40 cycles amplification: 95°C for 15 s and 60°C for 1 min, followed by a melt curve program: 95°C for 15 s, 60°C for 1min, and 95°C for

15 s. Relative differences in the expression of target genes were determined using the $2^{-\Delta\Delta CT}$ method [6,7].

Supplementary References

REFERENCES

1. Pezeshki, A., Fahim, A. & Chelikani, P.K. Dietary Whey and Casein Differentially Affect Energy Balance, Gut Hormones, Glucose Metabolism, and Taste Preference in Diet-Induced Obese Rats. *J Nutr* **145**, 2236-2244 (2015).
2. Hrabé de Angelis, M., Chambon, P. & Brown, S.D.M. *Standards of mouse model phenotyping*, (Wiley-VCH, Weinheim, 2006).
3. Nausheen, S., Shah, I.H., Pezeshki, A., Sigalet, D.L. & Chelikani, P.K. Effects of sleeve gastrectomy and ileal transposition, alone and in combination, on food intake, body weight, gut hormones, and glucose metabolism in rats. *Am.J.Physiol Endocrinol.Metab* **305**, E507-E518 (2013).
4. Wu, G. & Knabe, D.A. Free and protein-bound amino acids in sow's colostrum and milk. *J Nutr* **124**, 415-424 (1994).
5. Pezeshki, A. & Chelikani, P.K. Effects of Roux-en-Y gastric bypass and ileal transposition surgeries on glucose and lipid metabolism in skeletal muscle and liver. *Surg Obes Relat Dis* **10**, 217-228 (2014).
6. Livak, K.J. & Schmittgen, T.D. Analysis of relative gene expression data using real-time quantitative PCR and the 2(-Delta Delta C(T)) Method. *Methods* **25**, 402-408 (2001).
7. Schmittgen, T.D. & Livak, K.J. Analyzing real-time PCR data by the comparative C(T) method. *Nat.Protoc.* **3**, 1101-1108 (2008).
